# *N*-(9*H*-Fluoren-9-yl­idene)-4-methyl­aniline

**DOI:** 10.1107/S1600536809020765

**Published:** 2009-06-10

**Authors:** Su-Zhen Bai, Xin-Hua Lou, Hong-Mei Li, Hui Shi

**Affiliations:** a College of Chemistry and Chemical Engineering, Pingdingshan University, Pingdingshan 467002, People’s Republic of China; b College of Chemistry and Chemical Engineering, Luoyang Normal University, Luoyang 471022, People’s Republic of China; c Chemical Engineering and Pharmaceutics School, Henan University of Science and Technology, Luoyang 471003, People’s Republic of China

## Abstract

In the title compound, C_20_H_15_N, the fluorene unit is essentially planar [r.m.s. deviation 0.0334 Å] and the benzene ring bound to the imine N atom bears a methyl group which is nearly coplanar [dihedral angle 0.5 (1)°]. The dihedral angle between the substituent benzene ring and the 9*H*-fluoren-9-imine unit is 71.1 (3)°. Inter­molecular π–π inter­actions between the benzene rings of adjacent fluorene units [centroid–centroid distance 3.8081 (13) Å] are present in the crystal structure, resulting in a one-dimensional supra­molecular architecture.

## Related literature

For the properties of Schiff bases, see: Xu *et al.* (2007 ▶); Tanaka *et al.* (2006 ▶). For the properties of fluorene derivatives, see: Saragi *et al.* (2004 ▶). For related structures, see: Glagovich *et al.* (2004 ▶); Peters *et al.* (1998 ▶); Pierre *et al.* (1997 ▶).
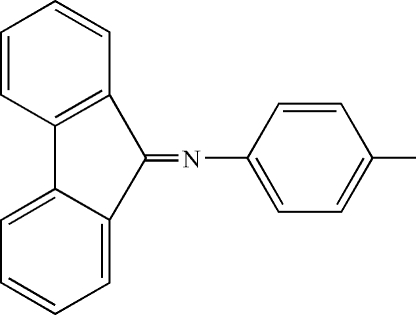


## Experimental

### 

#### Crystal data

C_20_H_15_N
*M* *_r_* = 269.33
Monoclinic, 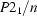

*a* = 5.6423 (10) Å
*b* = 12.187 (2) Å
*c* = 21.310 (4) Å
β = 94.441 (2)°
*V* = 1460.9 (5) Å^3^
*Z* = 4
Mo *K*α radiation
μ = 0.07 mm^−1^
*T* = 294 K
0.35 × 0.17 × 0.09 mm

#### Data collection

Bruker SMART APEX CCD area-detector diffractometer
Absorption correction: multi-scan (*SADABS*; Sheldrick, 1996 ▶) *T*_min_ = 0.976, *T*_max_ = 0.994
10793 measured reflections
2711 independent reflections
1779 reflections with *I* > 2σ(*I*)
*R*_int_ = 0.042

#### Refinement

*R*[*F* ^2^ > 2σ(*F* ^2^)] = 0.042
*wR*(*F* ^2^) = 0.115
*S* = 1.01
2711 reflections
192 parameters
H-atom parameters constrained
Δρ_max_ = 0.13 e Å^−3^
Δρ_min_ = −0.14 e Å^−3^

### 

Data collection: *SMART* (Bruker, 2004 ▶); cell refinement: *SAINT* (Bruker, 2004 ▶); data reduction: *SAINT*; program(s) used to solve structure: *SHELXS97* (Sheldrick, 2008 ▶); program(s) used to refine structure: *SHELXL97* (Sheldrick, 2008 ▶); molecular graphics: *SHELXTL* (Sheldrick, 2008 ▶); software used to prepare material for publication: *SHELXTL*.

## Supplementary Material

Crystal structure: contains datablocks global, I. DOI: 10.1107/S1600536809020765/si2179sup1.cif

Structure factors: contains datablocks I. DOI: 10.1107/S1600536809020765/si2179Isup2.hkl

Additional supplementary materials:  crystallographic information; 3D view; checkCIF report

## Figures and Tables

**Fig. 1.**
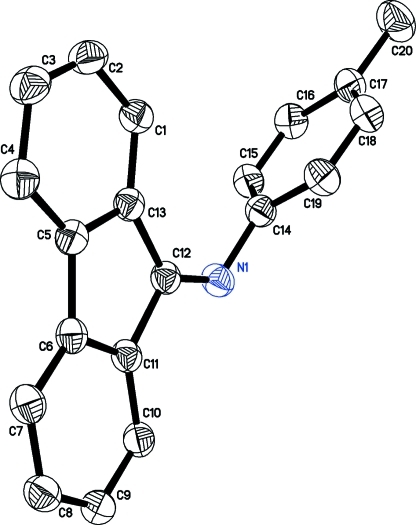
The molecular structure of the title compound with displacement ellipsoids at the 30% probability level. H atoms are omitted for clarity.

**Fig. 2.**
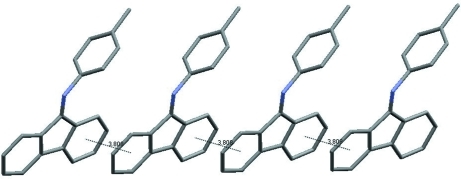
Partial view of the crystal packing showing the formation of the chain motif of molecules formed by the intermolecular π···π interactions. H atoms are omitted for clarity.

## supplementary crystallographic information

### Comment

Schiff bases have received much attention during the past decades because of their strong coordination capability and diverse biological activities (Xu *et al.*, 2007; Tanaka *et al.*, 2006). In addition, fluorene derivatives have found many applications in chemistry, especially in the optoelectronic area (Saragi *et al.*, 2004). In view of these important properties, the crystal structure of the title compond has been determined.

In the title compound (Fig.1), the C12—N1—C14 angle of 120.76 (15)° and the N1—C12 bond distance of 1.278 (2)Å are in close agreement with the similar *N*fluorenylideneaniline (Glagovich *et al.*, 2004; Peters *et al.*, 1998; Pierre *et al.*, 1997). The fluorene unit is essentially planar and the benzene ring bound to the imine N atom bears a methyl that is nearly coplanar. The dihedral angle between the substituent benzene ring and the 9*H*-fluoren-9-imine unit is 108.9 (3)°. Intermolecular π···π interactions between the benzene rings of adjacent fluorene units [centroid-centroid distance is 3.8081 (13) Å, the average perpendicular distance is 3.469 Å, the dihedral angle between the rings is 3.7°, symmetry code = -1 + *x*, *y*, *z*] are present in the crystal structure, resulting in a one-dimensional supramolecular architecture (Fig. 2).

### Experimental

The title compound was obtained from the condensation reaction of 9-fluorenone and 4-methylaniline as described in literature (Glagovich *et al.*, 2004) and recrystallized from ethanol solution at room temperature to give the desired product as yellow crystals suitable for single-crystal X-ray diffraction.

### Refinement

H atoms attached to C atoms of the title compound were placed in geometrically idealized positions and treated as riding with C—H distances constrained to 0.93–0.96 Å, and with *U*_iso_(H)=1.2*U*_eq_(C) (1.5*U*_eq_ for methyl H).

### Crystal data

**Table:**

C_20_H_15_N	*F*(000) = 568
*M**_r_* = 269.33	*D*_x_ = 1.225 Mg m^−^^3^
Monoclinic, *P*2_1_/*n*	Mo *K*α radiation, λ = 0.71073 Å
*a* = 5.6423 (10) Å	Cell parameters from 1911 reflections
*b* = 12.187 (2) Å	θ = 2.5–22.3°
*c* = 21.310 (4) Å	µ = 0.07 mm^−^^1^
β = 94.441 (2)°	*T* = 294 K
*V* = 1460.9 (5) Å^3^	Block, yellow
*Z* = 4	0.35 × 0.17 × 0.09 mm

### Data collection

**Table:**

Bruker SMART APEX CCD area-detector diffractometer	2711 independent reflections
Radiation source: fine-focus sealed tube	1779 reflections with *I* > 2σ(*I*)
graphite	*R*_int_ = 0.042
phi and ω scans	θ_max_ = 25.5°, θ_min_ = 2.5°
Absorption correction: multi-scan (*SADABS*; Sheldrick, 1996)	*h* = −6→6
*T*_min_ = 0.976, *T*_max_ = 0.994	*k* = −14→14
10793 measured reflections	*l* = −25→25

### Refinement

**Table:**

Refinement on *F*^2^	Secondary atom site location: difference Fourier map
Least-squares matrix: full	Hydrogen site location: inferred from neighbouring sites
*R*[*F*^2^ > 2σ(*F*^2^)] = 0.042	H-atom parameters constrained
*wR*(*F*^2^) = 0.115	*w* = 1/[σ^2^(*F*_o_^2^) + (0.052*P*)^2^ + 0.1725*P*] where *P* = (*F*_o_^2^ + 2*F*_c_^2^)/3
*S* = 1.00	(Δ/σ)_max_ < 0.001
2711 reflections	Δρ_max_ = 0.13 e Å^−^^3^
192 parameters	Δρ_min_ = −0.14 e Å^−^^3^
0 restraints	Extinction correction: *SHELXL*, Fc^*^=kFc[1+0.001xFc^2^λ^3^/sin(2θ)]^-1/4^
Primary atom site location: structure-invariant direct methods	Extinction coefficient: 0.0105 (19)

### Special details

**Table:**

Geometry. All e.s.d.'s (except the e.s.d. in the dihedral angle between two l.s. planes)are estimated using the full covariance matrix. The cell e.s.d.'s are takeninto account individually in the estimation of e.s.d.'s in distances, anglesand torsion angles; correlations between e.s.d.'s in cell parameters are onlyused when they are defined by crystal symmetry. An approximate (isotropic)treatment of cell e.s.d.'s is used for estimating e.s.d.'s involving l.s. planes.
Refinement. Refinement of *F*^2^ against ALL reflections. The weighted *R*-factor *wR* andgoodness of fit *S* are based on *F*^2^, conventional *R*-factors *R* are basedon *F*, with *F* set to zero for negative *F*^2^. The threshold expression of*F*^2^ > σ(*F*^2^) is used only for calculating *R*-factors(gt) *etc*. and isnot relevant to the choice of reflections for refinement. *R*-factors basedon *F*^2^ are statistically about twice as large as those based on *F*, and *R*-factors based on ALL data will be even larger.

### Fractional atomic coordinates and isotropic or equivalent isotropic displacement parameters (Å^2^)

**Table:**

	*x*	*y*	*z*	*U*_iso_*/*U*_eq_	
C1	0.0001 (3)	0.30235 (15)	0.13059 (9)	0.0507 (5)	
H1	−0.0938	0.3086	0.0929	0.061*	
C2	−0.0545 (4)	0.36053 (16)	0.18339 (10)	0.0582 (5)	
H2	−0.1869	0.4062	0.1810	0.070*	
C3	0.0836 (4)	0.35188 (16)	0.23918 (10)	0.0604 (5)	
H3	0.0452	0.3931	0.2737	0.072*	
C4	0.2787 (3)	0.28289 (15)	0.24478 (9)	0.0544 (5)	
H4	0.3701	0.2762	0.2828	0.065*	
C5	0.3348 (3)	0.22414 (13)	0.19248 (8)	0.0435 (4)	
C6	0.5230 (3)	0.14217 (13)	0.18583 (8)	0.0433 (4)	
C7	0.7076 (3)	0.10822 (14)	0.22712 (9)	0.0508 (5)	
H7	0.7270	0.1372	0.2676	0.061*	
C8	0.8643 (3)	0.03010 (15)	0.20743 (9)	0.0549 (5)	
H8	0.9901	0.0065	0.2349	0.066*	
C9	0.8350 (3)	−0.01288 (16)	0.14735 (9)	0.0557 (5)	
H9	0.9409	−0.0657	0.1350	0.067*	
C10	0.6513 (3)	0.02124 (14)	0.10530 (9)	0.0506 (5)	
H10	0.6328	−0.0077	0.0648	0.061*	
C11	0.4954 (3)	0.09953 (13)	0.12487 (8)	0.0432 (4)	
C12	0.2980 (3)	0.15766 (13)	0.08907 (8)	0.0440 (4)	
C13	0.1986 (3)	0.23442 (13)	0.13519 (8)	0.0428 (4)	
C14	0.0834 (3)	0.21043 (15)	−0.00479 (8)	0.0481 (5)	
C15	−0.1301 (3)	0.17101 (16)	−0.03143 (9)	0.0544 (5)	
H15	−0.1735	0.0986	−0.0246	0.065*	
C16	−0.2793 (4)	0.23849 (16)	−0.06821 (9)	0.0598 (5)	
H16	−0.4241	0.2110	−0.0852	0.072*	
C17	−0.2197 (4)	0.34635 (17)	−0.08065 (9)	0.0579 (5)	
C18	−0.0037 (4)	0.38372 (16)	−0.05457 (9)	0.0612 (5)	
H18	0.0417	0.4554	−0.0625	0.073*	
C19	0.1475 (4)	0.31784 (16)	−0.01699 (9)	0.0592 (5)	
H19	0.2921	0.3454	0.0001	0.071*	
C20	−0.3829 (4)	0.4195 (2)	−0.12150 (12)	0.0880 (8)	
H20A	−0.3234	0.4263	−0.1623	0.132*	
H20B	−0.3899	0.4907	−0.1024	0.132*	
H20C	−0.5393	0.3880	−0.1257	0.132*	
N1	0.2459 (3)	0.14086 (12)	0.03045 (7)	0.0521 (4)	

### Atomic displacement parameters (Å^2^)

**Table:**

	*U*^11^	*U*^22^	*U*^33^	*U*^12^	*U*^13^	*U*^23^
C1	0.0542 (11)	0.0462 (10)	0.0518 (11)	0.0015 (9)	0.0048 (9)	0.0011 (9)
C2	0.0594 (12)	0.0508 (11)	0.0659 (14)	0.0066 (9)	0.0144 (11)	−0.0045 (10)
C3	0.0717 (14)	0.0513 (12)	0.0600 (13)	0.0007 (10)	0.0164 (11)	−0.0119 (10)
C4	0.0629 (12)	0.0494 (11)	0.0507 (11)	−0.0054 (9)	0.0037 (9)	−0.0071 (9)
C5	0.0486 (10)	0.0379 (9)	0.0443 (10)	−0.0061 (8)	0.0062 (8)	−0.0038 (8)
C6	0.0477 (10)	0.0395 (9)	0.0429 (10)	−0.0065 (8)	0.0040 (8)	0.0014 (8)
C7	0.0565 (11)	0.0494 (11)	0.0457 (11)	−0.0039 (9)	−0.0005 (9)	0.0003 (9)
C8	0.0497 (11)	0.0555 (12)	0.0587 (13)	0.0015 (9)	−0.0008 (10)	0.0083 (10)
C9	0.0585 (12)	0.0521 (11)	0.0578 (13)	0.0085 (9)	0.0123 (10)	0.0071 (10)
C10	0.0630 (12)	0.0441 (10)	0.0455 (11)	0.0034 (9)	0.0102 (9)	0.0023 (8)
C11	0.0497 (10)	0.0365 (9)	0.0438 (10)	−0.0017 (8)	0.0053 (8)	0.0022 (8)
C12	0.0509 (11)	0.0381 (9)	0.0434 (11)	−0.0027 (8)	0.0064 (8)	0.0007 (8)
C13	0.0494 (10)	0.0350 (9)	0.0446 (10)	−0.0043 (8)	0.0075 (8)	−0.0004 (8)
C14	0.0591 (12)	0.0486 (10)	0.0366 (10)	0.0046 (9)	0.0029 (9)	−0.0027 (8)
C15	0.0632 (12)	0.0498 (11)	0.0502 (11)	−0.0031 (10)	0.0038 (10)	0.0021 (9)
C16	0.0562 (12)	0.0647 (13)	0.0572 (12)	−0.0059 (10)	−0.0030 (10)	0.0009 (10)
C17	0.0627 (13)	0.0626 (13)	0.0478 (11)	0.0034 (10)	0.0006 (10)	0.0082 (10)
C18	0.0700 (14)	0.0521 (12)	0.0610 (13)	−0.0019 (10)	0.0020 (11)	0.0089 (10)
C19	0.0609 (12)	0.0554 (12)	0.0597 (13)	−0.0027 (10)	−0.0046 (10)	0.0022 (10)
C20	0.0844 (17)	0.0904 (18)	0.0862 (18)	0.0081 (14)	−0.0126 (14)	0.0261 (14)
N1	0.0638 (10)	0.0489 (9)	0.0431 (9)	0.0066 (8)	0.0002 (8)	−0.0012 (7)

### Geometric parameters (Å, °)

**Table:**

C1—C2	1.385 (3)	C10—H10	0.9300
C1—C13	1.390 (2)	C11—C12	1.481 (2)
C1—H1	0.9300	C12—N1	1.278 (2)
C2—C3	1.374 (3)	C12—C13	1.497 (2)
C2—H2	0.9300	C14—C15	1.378 (2)
C3—C4	1.383 (3)	C14—C19	1.388 (3)
C3—H3	0.9300	C14—N1	1.420 (2)
C4—C5	1.382 (2)	C15—C16	1.378 (3)
C4—H4	0.9300	C15—H15	0.9300
C5—C13	1.397 (2)	C16—C17	1.387 (3)
C5—C6	1.473 (2)	C16—H16	0.9300
C6—C7	1.374 (2)	C17—C18	1.377 (3)
C6—C11	1.397 (2)	C17—C20	1.509 (3)
C7—C8	1.386 (3)	C18—C19	1.381 (3)
C7—H7	0.9300	C18—H18	0.9300
C8—C9	1.381 (3)	C19—H19	0.9300
C8—H8	0.9300	C20—H20A	0.9600
C9—C10	1.381 (2)	C20—H20B	0.9600
C9—H9	0.9300	C20—H20C	0.9600
C10—C11	1.384 (2)		
			
C2—C1—C13	118.45 (18)	C6—C11—C12	109.05 (15)
C2—C1—H1	120.8	N1—C12—C11	122.27 (16)
C13—C1—H1	120.8	N1—C12—C13	132.28 (16)
C3—C2—C1	121.13 (18)	C11—C12—C13	105.42 (14)
C3—C2—H2	119.4	C1—C13—C5	120.05 (16)
C1—C2—H2	119.4	C1—C13—C12	131.81 (16)
C2—C3—C4	121.03 (18)	C5—C13—C12	108.00 (15)
C2—C3—H3	119.5	C15—C14—C19	118.94 (17)
C4—C3—H3	119.5	C15—C14—N1	121.18 (17)
C5—C4—C3	118.41 (18)	C19—C14—N1	119.68 (17)
C5—C4—H4	120.8	C16—C15—C14	120.18 (18)
C3—C4—H4	120.8	C16—C15—H15	119.9
C4—C5—C13	120.91 (17)	C14—C15—H15	119.9
C4—C5—C6	129.85 (17)	C15—C16—C17	121.85 (19)
C13—C5—C6	109.18 (15)	C15—C16—H16	119.1
C7—C6—C11	120.53 (16)	C17—C16—H16	119.1
C7—C6—C5	131.23 (16)	C18—C17—C16	117.14 (18)
C11—C6—C5	108.22 (15)	C18—C17—C20	121.3 (2)
C6—C7—C8	118.85 (18)	C16—C17—C20	121.6 (2)
C6—C7—H7	120.6	C17—C18—C19	121.97 (19)
C8—C7—H7	120.6	C17—C18—H18	119.0
C9—C8—C7	120.51 (18)	C19—C18—H18	119.0
C9—C8—H8	119.7	C18—C19—C14	119.90 (18)
C7—C8—H8	119.7	C18—C19—H19	120.1
C10—C9—C8	121.14 (18)	C14—C19—H19	120.1
C10—C9—H9	119.4	C17—C20—H20A	109.5
C8—C9—H9	119.4	C17—C20—H20B	109.5
C9—C10—C11	118.36 (17)	H20A—C20—H20B	109.5
C9—C10—H10	120.8	C17—C20—H20C	109.5
C11—C10—H10	120.8	H20A—C20—H20C	109.5
C10—C11—C6	120.60 (17)	H20B—C20—H20C	109.5
C10—C11—C12	130.17 (16)	C12—N1—C14	120.76 (15)
			
C13—C1—C2—C3	0.1 (3)	C2—C1—C13—C5	1.4 (3)
C1—C2—C3—C4	−1.4 (3)	C2—C1—C13—C12	176.50 (17)
C2—C3—C4—C5	1.2 (3)	C4—C5—C13—C1	−1.6 (3)
C3—C4—C5—C13	0.3 (3)	C6—C5—C13—C1	176.08 (15)
C3—C4—C5—C6	−176.88 (17)	C4—C5—C13—C12	−177.78 (15)
C4—C5—C6—C7	−6.8 (3)	C6—C5—C13—C12	−0.07 (18)
C13—C5—C6—C7	175.76 (17)	N1—C12—C13—C1	8.8 (3)
C4—C5—C6—C11	175.26 (18)	C11—C12—C13—C1	−173.40 (17)
C13—C5—C6—C11	−2.18 (18)	N1—C12—C13—C5	−175.62 (18)
C11—C6—C7—C8	−0.7 (3)	C11—C12—C13—C5	2.14 (17)
C5—C6—C7—C8	−178.41 (17)	C19—C14—C15—C16	−1.7 (3)
C6—C7—C8—C9	−0.1 (3)	N1—C14—C15—C16	−176.54 (17)
C7—C8—C9—C10	0.6 (3)	C14—C15—C16—C17	1.2 (3)
C8—C9—C10—C11	−0.3 (3)	C15—C16—C17—C18	0.0 (3)
C9—C10—C11—C6	−0.4 (3)	C15—C16—C17—C20	179.4 (2)
C9—C10—C11—C12	174.14 (17)	C16—C17—C18—C19	−0.7 (3)
C7—C6—C11—C10	1.0 (3)	C20—C17—C18—C19	179.9 (2)
C5—C6—C11—C10	179.15 (15)	C17—C18—C19—C14	0.2 (3)
C7—C6—C11—C12	−174.66 (15)	C15—C14—C19—C18	1.0 (3)
C5—C6—C11—C12	3.53 (18)	N1—C14—C19—C18	175.92 (17)
C10—C11—C12—N1	−0.5 (3)	C11—C12—N1—C14	−169.33 (16)
C6—C11—C12—N1	174.52 (16)	C13—C12—N1—C14	8.1 (3)
C10—C11—C12—C13	−178.57 (17)	C15—C14—N1—C12	−115.92 (19)
C6—C11—C12—C13	−3.52 (18)	C19—C14—N1—C12	69.2 (2)
